# Risk stratification by endocrinologists of patients with type 2 diabetes in a Danish specialised outpatient clinic: a cross-sectional study

**DOI:** 10.1186/s12913-016-1365-y

**Published:** 2016-04-09

**Authors:** Lene Munch, Anne B. Arreskov, Michael Sperling, Dorthe Overgaard, Filip K. Knop, Tina Vilsbøll, Michael E. Røder

**Affiliations:** Center for Diabetes Research, Gentofte Hospital, University of Copenhagen, Kildegårdsvej 28, DK-2900 Hellerup, Denmark; Institute of Nursing, University College Metropol, Tagensvej 86, DK-2200 Copenhagen, Denmark; The SAS Institute, Copenhagen, Denmark; NNF Center for Basic Metabolic Research and Department of Biomedical Sciences, Faculty of Health and Medical Sciences, University of Copenhagen, Blegdamsvej 3, DK-2200 Copenhagen, Denmark

**Keywords:** Organising diabetes care, Outpatient clinic, Risk assessment, Risk stratification, Type 2 diabetes

## Abstract

**Background:**

To target optimised medical care the Danish guidelines for diabetes recommend stratification of patients with type 2 diabetes (T2D) into three levels according to risk and complexity of treatment. The aim was to describe the T2D population in an outpatient clinic, measure the compliance of the endocrinologists’ to perform risk stratification, and investigate the level of concordance between stratification performed by the endocrinologists and objective assessments.

**Methods:**

A cross-sectional study with data collected from medical records and laboratory databases. The Danish risk stratification model contained the following criteria: HbA_1c_, blood pressure, metabolic complications, microvascular and macrovascular complications. Stratification levels encompassed: level 1 (uncomplicated), level 2 (intermediate risk) and level 3 (high risk). Objective assessments were conducted independently by two health professionals, and compared with the endocrinologists’ assessments. In order to test the degree of concordance, we conducted Cohen's kappa, McNemar’s test for marginal homogeneity, and Bowker’s test for symmetry.

**Results:**

Of 245 newly referred patients, 209 (85 %) were stratified by the endocrinologists to level 1 (16 %), level 2 (55 %) and level 3 (29 %). By objective assessments, 4 % were stratified to level 1, 51 % to level 2 and 45 % to level 3. Of 419 long-term follow-up patients, 380 (91 %) were stratified by the endocrinologists to level 1 (5 %), level 2 (57 %), level 3 (38 %). By objective assessments, 3 % were stratified to level 1, 58 % to level 2 and 39 % to level 3. The concordance rate between endocrinologists’ and objective assessments was 63 % among newly referred (kappa 0.39; fair agreement) and 67 % for long-term follow-up (kappa 0.45; moderate agreement). Among newly referred patients, the endocrinologists stratified less patients at level 3 compared to objective assessments (*p* < 0.0001). There were no significant differences in marginal distribution within long-term follow-up patients.

**Conclusion:**

Type 2 diabetes patients, newly referred to or allocated for long-term follow-up in the out-patient clinic, were mainly intermediate and high-risk, complicated patients (96 % and 95 %, respectively). Compliance of stratification by endocrinologists was high. The concordance between endocrinologists’ and objective assessments was not strong. Our data suggest that clinician-support for stratification level categorisation might be needed.

## Background

The global prevalence of type 2 diabetes (T2D) is increasing [1–3]. The number of adults with diabetes was estimated to be 382 million people in 2013 and rising to 592 million people in 2035 [2, 3]. T2D is a multifactorial and chronic disease associated with serious complications and co-morbidities, and in 2014 it was ranked as the 8^th^ leading cause of deaths worldwide [4] and predicted to increase to the 7^th^ ranking in 2030 [5]. Patients with T2D have an increased morbidity and mortality compared to the general population [6–8] due to an increased risk of macrovascular disease as well as microvascular complications such as nephropathy, retinopathy and neuropathy. Studies have shown that the risk of complications associated with T2D can be reduced or delayed among patients with newly diagnosed T2D by initiating and keeping tight glucose [9] and blood pressure control [10]. In the Steno-2 study patients with T2D and micro-albuminuria were offered an intensive multifactorial intervention, and demonstrated significant reductions in micro and macrovascular diseases at 13.3 years-follow-up compared to patients in conventional therapy [11]. Thus, in order to prevent or delay the onset of complications, it is essential to offer an evidence-based and well-organised diabetes care.

Taking the growing number of patients with T2D into account, it is relevant to target allocation of medical care according to the need of the individual patient and the existing resources [12]. In order to meet these circumstances the Danish National Board of Health integrated elements of the Chronic Care Model [13, 14] and risk stratification in a national strategy for treatment and care of patients with chronic diseases in 2008 [15]. As a consequence, the Capital Region of Denmark developed a program for patients with T2D, recommending patients with T2D to be stratified into three levels depending on the severity and complications of their disease [16]. Stratification may be used to objectively assess and target patients to treatment, care and specialty level according to the severity of the disease and the patient's ability to self-care.

Patients stratified to level 1 have well-controlled T2D without clinically relevant complications, while patients at level 2 and level 3 have less well controlled T2D and/or increasing severity of complications and/or co-morbidity. According to the model, patients stratified to level 3 should be followed in hospital-based outpatient clinics where specialised and multidisciplinary treatment is available [16, 17]. Patients at level 1 are assumed to be allocated to follow-up in general practice while patients at level 2 are recommended intensified control by either the general practitioner or the outpatient clinic, or ideally, in cooperation between general practice and outpatient clinic, preferably in a shared care follow-up [16].

The Danish risk stratification model may be a useful tool in managing diabetes care for the growing number of patients with T2D, as the model can offer guidance in the distribution of both economic resources and the care burden between general practitioners and outpatient clinics. To what extent the Danish risk stratification model is used in general practices or in hospitals, is currently unknown. In our outpatient clinic local instructions recommend that risk stratification takes place at the initial medical examination, at discharge from the diabetes clinic or at the transition to long-term follow-up, as well as once a year at the annual extensive check-up.

The purpose of this study was to describe the patient population according to stratification levels in a hospital-based diabetes outpatient clinic and to investigate the compliance of the endocrinologists to perform stratification in accordance with an established risk stratification model. Furthermore, we wanted to test the concordance between the assessment performed by the endocrinologists, and the objective assessment by the established model available to the clinician.

## Methods

We included patients with T2D followed in a hospital-based diabetes outpatient clinic (Gentofte Hospital, University of Copenhagen, Denmark). Patients included in the trial were those followed in the clinic during a period of 15 month (January 2013 until March 2014). Patients were identified from the electronic records and were included if they had T2D and had an initial medical examination (newly referred patients) or an annual extensive check-up (long-term follow-up patients).

### Patients

Patients attending the clinic were divided into either newly referred or long-term follow-up patients. The newly referred patients underwent initial medical examination and attended medical visits during a period of approximately six months. After this initial period the patients were either discharged to be followed by their general practitioner or transferred to long-term follow-up in the diabetes outpatient clinic based upon their level of stratification. Patients in long-term follow-up were offered a minimum of four visits a year; two control visits with an endocrinologist, one with a trained diabetes nurse, and an annual extensive check-up with an endocrinologist. Both the initial examination and the annual extensive check-up included blood and urine sampling, ECG, fundus photography, as well as consultation with and risk stratification by an endocrinologist. We included patients with T2D. The patients were diagnosed with T2D before referral to the outpatient clinic due to elevated HbA1c according to international guidelines. Furthermore, all patients had measurable C-peptide levels and/or long-term treatment with oral antidiabetic drugs. Patients with secondary diabetes and type 1 diabetes were excluded.

### Data collection

**Fig. 1:**
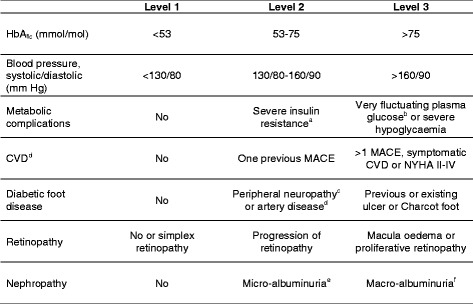
The Danish risk stratification model for patients with type 2 diabetes. All parameters in level 1 have to be fulfilled to be allocated to risk stratification level 1. At risk stratification level 2 at least one parameter has to be fulfilled in level 2, and none in level 3. Patients at level 3 have to fulfil at least one of the parameters in level 3 [16]. HbA_1c_, haemoglobin A_1c_; CVD, cardiovascular disease; MACE, major cardiovascular event; NYHA, the New York Heart Association functional classification in patients with heart disease [38]. ^a^ Severe insulin resistance: Insulin dose > 2.0 U/kg/day. ^b^ Very fluctuating plasma glucose: Daily plasma glucose values of >15 mmol/l or <5 mmol/l. ^c^ Peripheral neuropathy: Vibration perception threshold ≥25 mV evaluated by a biothesiometer. ^d^ Peripheral artery disease: Ankle-brachial index <0.9 with or without symptomatic claudication. ^e^ Micro-albuminuria: >1 occasion of urine-albumin/creatinine ratio between 30 and 300 mg/g. ^f^ Macro-albuminuria:^:^ Urine-albumin/creatinine ratio ≥300 mg/g or an estimated glomerular filtration rate <60 ml/min

The Danish risk stratification model stratifies patients into three levels by their level of HbA1c, blood pressure, and presence of albuminuria, as well as the presence of micro- and macrovascular complications. The model is designed as an organizational tool intending to graduate specialised care and treatment according to the severity and complexity of the disease. The model was originally compiled by diabetes specialists and defined in a report by the Capital Region of Denmark [16, 17]. The thresholds and definitions characterising the three levels were constructed by means of current evidence [16, 18–21]. In the present study, the model was slightly modified in order to clarify the criteria and make the model more operational, however not altering the overall model criteria. The criteria for the different levels in the model used are shown in Fig. 1. No formal clinical definition of severe insulin resistance exists in the literature to our knowledge. In our study, severe insulin resistance was defined as an insulin requirement >2.0 U/kg/day. This was based on a mean dose of insulin required for treatment of normal weight younger people with type 1 diabetes (assumed to be insulin sensitive) of approximately 0.6 U/kg/day [22]. Severe insulin resistance was set to an insulin sensitivity below 30 % of the mean normal level, corresponding to an insulin dose above 2.0 U/kg/day. All criteria have to be fulfilled to be allocated to level 1, only one criteria have to be fulfilled to be allocated to level 3. Patients not fulfilling criteria for level 1 or 3 were allocated to level 2.

Objective stratification was conducted by applying the Danish risk stratification model to the collected computerised data. Data were collected from medical records, laboratory files and the Clinical System Organizer/DiabetesRask. The latter being a specific diabetes database containing all diabetes relevant data regarding treatment and status of the individual patient. All data concerning the newly referred patients were identified at the first initial visit and data concerning the long-term follow-up patients were identified at the most recent annual extensive check-up. For the long-term follow-up patients, the results of urine and blood samples were identified at the most recent visit at the outpatient clinic in the study period. Two authors; Lene Munch (LM) and Michael E. Røder (MR) conducted the objective stratification independently. In case of disagreement between the objective assessments, the results were re-examined by LM and MR in order to reach consensus. The model used was identical to the one available to the endocrinologists in the outpatient clinic. In case of missing data, patients were stratified according to the data available. Among the newly referred patients, 17 patients had missing data according to the seven parameters in the risk stratification model; in 11 patients one missing and in six patient two missing data parameters in the risk stratification model. Among the long-term follow-up patients 20 had one missing data parameter.

We aimed to explore plausible reasons for disagreement between the endocrinologists’ and the objective assessments. In cases, where the objective stratification level was higher than the assessment performed by the endocrinologist, the reason for higher assessment was registered according to the seven parameters in the Danish risk stratification model (Fig. 1). In cases, where the endocrinologist was assessing the patient to a higher level than the objective assessment, plausible reasons for the endocrinologist’s assessment was estimated on the basis of variables concerning co-morbidity, body mass index (BMI) >35 kg/m^2^ and vulnerability, or no known reason.

Reporting of data results was made in accordance to the STROBE statement checklist.

### Statistical methods

In the description of the population’s clinical characteristics and disease status, both newly referred and long-term follow-up patients were categorised into risk stratification levels, as it was assessed by the objective assessors. Data are presented as mean ± standard deviation (SD) and frequency and percentages for ordinal and nominal data. We wanted to investigate possible associations between the objective assessment and whether or not the patients were risk stratified by the endocrinologist. This was tested by conducting Chi-square tests.

To measure the concordance between the clinical and objective assessments, we used Cohen's kappa [23]. As data were ordinal we conducted a weighted kappa, which accounted for the size of disagreement. The following defined the strength of agreement for the kappa coefficient: 0 = poor, 0.01–0.20 = slight, 0.21–0.40 = fair, 0.41–0.60 = moderate, 0.61–0.80 = substantial, and 0.81–1 = almost perfect [24]. Furthermore, we used McNemar’s test to test for marginal homogeneity [25], and Bowker’s test to test for symmetry above and below the main diagonal [26].

*P* values of less than 0.05 were considered significant. Data were analysed using SAS Enterprise guide, version 5.2.

## Ethics

The study was conducted in accordance with the principles of the Helsinki declaration. The Danish Data Protection Agency approved the study protocol, anonymity of the participants, the protection of identity, privacy and handling of the data (journal no. 2007-58-0015).

## Availability of Supporting Data

The database set was available for all authors of the study, and will be available for other non-commercial researchers on request.

## Results

A total of 946 patients were identified and 21 were excluded; one patient never had an appointment and was referred to another clinic and 20 patients were referred to our clinic in March 2014, but did not have any appointments until after the study period.

### Sample characteristics

**Fig. 2:**
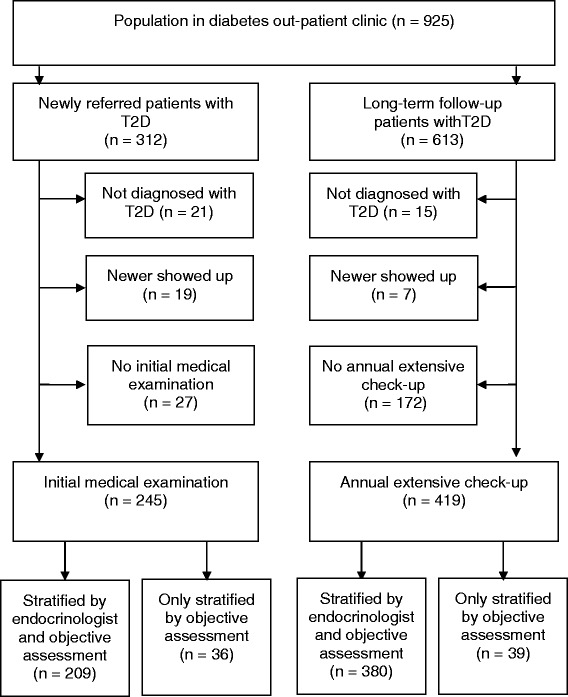
Disposition of study population. T2D, type 2 diabetes

The population in the outpatient diabetes clinic consisted of 925 patients with T2D. Of these 664 (72 %) were included in the study. Twenty-two percent of the population in the clinic did not have an initial medical examination or an annual extensive check-up performed during the period (Fig. 2). Demographic and clinical characteristics of the newly referred and the long-term follow-up patients are presented in Table 1.

**Table 1:** Demographic and clinical characteristics of the newly referred and long-term follow-up patients according to objective stratification levels

	Newly referred patients				Long-term follow-up patients			
	Level 1 (*n* = 9)	Level 2 (*n* = 125)	Level 3 (*n* = 111)	All (*n* = 245)	Level 1 (*n* = 18)	Level 2 (*n* = 236)	Level 3 (*n* = 165)	All (*n* = 419)
Age (years, range)	55.3 (42–62)	62.3 (23–89)	65.8 (23–93)	63.6 (23–93)	55.3 (28–68)	63.6 (19–89)	68.6 (38–93)	65.2 (19–93)
Male sex – no. (%)	3 (33.3)	82 (65.6)	71 (64.0)	156 (63.7)	9 (50)	160 (67.8)	113 (68.5)	282 (67.3)
BMI (kg/m2)a	30.3 ± 8.2	30.5 ± 6.2	29.6 ± 5.3	30.1 ± 6.1	28.0 ± 5.3	30.1 ± 5.6	30.5 ± 5.9	30.2 ± 5.7
Diabetes duration - years	3.9 ± 5.1	5.4 ± 5.8	7.9 ± 8.0	6.5 ± 7.0	6.2 ± 5.3	8.5 ± 6.0	11.5 ± 7.1	9.6 ± 6.6
SBP (mmHg)	120.6 ± 7.0	140.6 ± 15.8	142.3 ± 24.4	140.7 ± 19.8	114.8 ± 9.1	134.0 ± 13.8	136.7 ± 17.4	134.3 ± 15.7
DBP (mmHg)	70.9 ± 5.2	81.1 ± 9.5	80.0 ± 12.7	80.3 ± 11.1	72.3 ± 5.3	78.2 ± 8.7	76.8 ± 10.1	77.4 ± 9.2
HbA1c (%) (mmol/mol)	6.3 ± 2.4 (45 ± 3)	7.1 ± 3.1 (54 ± 10)	8.5 ± 4.5 (69 ± 26)	7.7 ± 4.1 (61 ± 21)	6.1 ± 2.7 (43 ± 6)	6.8 ± 3.1 (51 ± 10)	7.6 ± 3.9 (60 ± 19)	7.1 ± 3.5 (54 ± 15)
TC (mmol/l)	4.8 ± 0.8	4.4 ± 1.0	4.3 ± 1.2	4.3 ± 1.1	4.2 ± 1.4	4.0 ± 0.8	4.1 ± 1.1	4.0 ± 0.9
LDL-C (mmol/l)	2.3 ± 0.7	2.2 ± 1.0	2.1 ± 0.9	2.2 ± 0.9	2.2 ± 1.1	1.8 ± 0.6	1.9 ± 1.0	1.8 ± 0.8
HDL-C(mmol/l)	1.7 ± 0.4	1.3 ± 0.5	1.2 ± 0.4	1.3 ± 0.5	1.4 ± 0.4	1.3 ± 0.4	1.3 ± 0.4	1.3 ± 0.4
TG (mmol/l)	1.9 ± 0.6	2.0 ± 1.2	2.3 ± 1.5	2.1 ± 1.3	1.2 ± 0.6	2.1 ± 1.2	2.2 ± 1.3	2.1 ± 1.2
Retinopathy – no. (%)								
Any stage	0	3 (2.4)	6 (5.4)	9 (3.7)	0	20 (8.5)	35 (21.2)	55 (13.1)
Macular oedema or								
proliferative retinopathy	0	0	3 (2.7)	3 (1.2)	0	0	4 (2.4)	4 (1.0)
Neuropathy – no. (%)								
Peripheral neuropathy or insufficiency	0	60 (48.0)	58 (52.3)	118 (48.2)	0	128 (54.2)	111 (67.3)	139 (33.2)
Previous or existing ulcer or Charcot foot	0	0	8 (7.2)	8 (3.3)	0	0	13 (7.9)	13 (3.1)
Nephropathy – no. (%)	0	0	36 (32.4)	36 (14.7)	0	0	89 (53.9)	89 (21.2)
Former MACE – no. (%)	0	25 (20.0)	34 (30.6)	59 (24.1)	0	45 (19.1)	51 (30.9)	96 (22.9)
>1 MACE, symptomatic CVD or NYHA II-IV – no. (%)	0	0	26 (23.4)	26 (10.6)	0	0	56 (33.9)	56 (13.4)

Mean ± standard deviation

*BMI* body mass index; *SBP* systolic blood pressure; *DBP* diastolic blood pressure; *HbA1c* haemoglobin A1c; *TC* total cholesterol; *LDL* low density lipoprotein; *HDL* high density lipoprotein; *TG* triglycerides; *CVD* cardiovascular disease; *MACE* major cardiovascular event; *NYHA* the New York Heart Association functional classification in patients with heart disease [36]

### Compliance to risk stratification

In total 664 patients had an initial medical examination or an annual extensive check-up and of these, 589 (89 %) patients were risk stratified. Among the newly referred patients 245 had an initial medical examination, and 209 (85 %) of these patients were risk stratified by endocrinologists. Of the 419 patients in long-term follow-up, 380 (91 %) were risk stratified by an endocrinologist. For the newly referred patients there was no difference in whether or not patients were risk stratified by the endocrinologists compared to the levels of objective assessments (*p* = NS) (Table 2). Among the long-term follow-up patients there was a significant difference in whether or not patients were risk stratified by the endocrinologists compared to the levels of objective assessments, as more patients at level 1 (33 %), compared to patients at level 2 (7 %) or 3 (10 %), were not risk stratified by the endocrinologists (*p* < 0.001) (Table 2).

**Table 2:** Possible associations between risk stratification levels by objective assessment and whether or not patients were stratified by the endocrinologist

Newly referred patients – *n* (%)				
		Objective assessment		
		Level 1	Level 2	Level 3
Assessment by endocrinologist	No	0 (0)	19 (15)	17 (15)
	Yes	9 (100)	106 (85)	94 (85)
	Total	9 (100)	125 (100)	111 (100)
Chi-Square test: *p* = NS				
Long-term follow-up patients – *n* (%)				
		Objective assessment		
		Level 1	Level 2	Level 3
Assessment by endocrinologist	No	6 (33)	16 (7)	17 (10)
	Yes	12 (67)	220 (93)	148 (90)
	Total	18 (100)	236 (100)	165 (100)
Chi-Square test: *p* < 0.001				

### Concordance in risk stratification

The rates of concordance are illustrated in Table 3. Among the newly referred patients there was a fair agreement between the assessments conducted by the endocrinologists and the objective assessments (kappa 0.39). There was a difference in the marginal distribution, as the endocrinologists categorised significantly less patients at level 3 compared to the objective assessment (*p* < 0.0001) (Table 3). Within the group of newly referred patients, there were more cases of disagreement due to the objective assessment being higher than the endocrinologists’ assessment (83 %) compared to cases where the endocrinologist assessed higher than the objective assessment (17 %) (*p* < 0.0001). The two most frequent reasons for the objective assessments being higher than the endocrinologists’ assessments were when the values of HbA_1c_ (*N* = 32 (49 %)) and blood pressure (*N* = 25 (38 %)) were not taken into account by the endocrinologist. Due to few cases of the endocrinologists assessing higher than the objective assessment, there were no patterns in the reasons for these mismatches.

**Table 3:** Concordance of endocrinologists´ and objective assessment of risk stratification in newly referred and in long-term follow-up patients

Newly referred patients – *n* (%)					
		Objective assessment			
		Level 1	Level 2	Level 3	Total
Endocrinologists´ assessment	Level 1	9 (4.3)	18 (8.6)	7 (3.3)	34 (16.3)
	Level 2	0	75 (36.9)	40 (19.1)	115 (55.0)
	Level 3	0	13 (6.2)	47 (22.5)	60 (28.7)
	Total	9 (4.3)	106 (50.7)	94 (45.0)	209 (100)
Observed agreement: 62.7 %, kappa = 0.39 (CI: 0.29–0.50), McNemar’s test for marginal distribution: *p* < 0.0001, Bowker’s test for symmetry: *p* < 0.0001					
Long-term follow-up patients – *n* (%)					
		Objective assessment			
		Level 1	Level 2	Level 3	Total
Endocrinologists´ assessment	Level 1	7 (1.8)	12 (3.2)	1 (0.3)	20 (5.3)
	Level 2	5 (1.3)	162 (42.6)	51 (13.4)	218 (57.4)
	Level 3	0	46 (12.1)	96 (25.38)	142 (37.4)
	Total	12 (3.2)	220 (57.9)	148 (38.9)	380 (100)
Observed agreement: 69.7 %, kappa = 0.45 (CI: 0.36–0.53), McNemar’s test for marginal distribution: *p* = NS, Bowker’s test for symmetry: *p* = NS					

*CI* confidence interval

Among the patients in long-term follow-up, there was agreement in approximately 2/3 of cases, corresponding to a moderate concordance (kappa 0.45). The test for marginal distribution found consistency in the proportion of stratification levels between the endocrinologists’ and the objective assessments (*p* = NS) (Table 3). The cases of disagreement were equally distributed above (56 %) and below (44 %) the main diagonal (NS). The two most frequent reasons for the objective assessment being higher than the endocrinologist’s assessment were when the values of nephropathy (*N* = 20 (31 %)) and HbA_1c_ (*N* = 16 (25 %)) were not taken into account by the endocrinologist. The most frequent plausible reason for endocrinologist assessing higher than the objective assessment was the presence of co-morbidity (*N* = 35 (69 %)).

## Discussion

In the present study we found that the vast majority of the patients with T2D in a hospital-based diabetes outpatient clinic were allocated to stratification level 2 or 3 (96 %), and only 4 % were allocated to level 1. The compliance of stratification performance by the endocrinologists was quite high, with 9 out of 10 of patients attending the clinic being risk stratified. Disagreement between the endocrinologists’ assessments and the objective assessments was found in a number of cases, corresponding to the level of concordance being evaluated as fair among the newly referred and moderate among patients in long-term follow-up. The disagreements among the newly referred patients were primarily due to the objective assessments being higher than the endocrinologists’. Among the patients in long-term follow-up the mismatches were almost equally distributed between the objective and the endocrinologists’ assessments.

The proportion of the three levels of risk stratification is often represented as a pyramid shape in the context of the chronic care model with most patients in level 1, and fewest in level 3 [13, 27, 28]. A Dutch study testing a disease management program for patients with diabetes (97 % with T2D) found a pyramid-shaped distribution of the patients at baseline (Low complexity: 54 %, Medium complexity: 34 %, High complexity: 10 %) [28]. Their definitions of the three levels were however not very detailed, but overall it seems comparable to the model we used. The patients were recruited from both general practitioners and an outpatient clinic, and the group allocation was performed by a team, consisting of the general practitioner, a diabetes nurse specialist and an endocrinologist. However, after a 24-month follow-up period the distribution of patients had changed to a non-pyramid shape, as 66 % of the patients were assigned to the medium complexity group (level 2), while 23 % and 11 % were assigned to low (level 1) and high (level 3) complexity, respectively [28]. The change in the size of the groups was mainly due to changes in HbA_1c_ in the patients at level 1 at follow-up, which lead to many patients being transferred from level 1 to level 2 [28]. The predominance of patients in level 2 were in line with a Danish study [29], using a risk stratification model similar to ours. They identified patients with diabetes, primarily T2D, followed by either a general practitioner or at a specialised outpatient clinic, via medical records from general practitioners. Sixty-two percent of these patients were stratified to level 2, while level 1 and 3 accounted for 21 % and 15 %, respectively [29]. The distribution of patients’ level of risk stratification was not pyramid-shaped in our study, as the vast majority of patients were level 2 or 3 patients. This is in line with two Asian studies stratifying ambulatory T2D patients, into four risk levels, as 59 % [30] and 64 % [31] of the patients were categorised into the high risk level (level 3). The population distribution in our study is in accordance with guideline recommendations allocating patients with a substantial degree of risk and disease complexity to specialised hospital-based diabetes outpatient clinics, and patients at risk level 1 should solely be managed by the general practitioner [16]. Whether or not the population distribution of risk stratification will appear as a pyramid shape may depend on the criteria defining the three levels. According to Kaiser-Permanente, who developed the three-level risk stratification model, the threshold value for HbA_1c_ for level 2 was 10 % [13], while it was 7 % in the risk stratification model used in our study and the study by Qvist et al. [29].

One possible reason for the discrepancy between assessments conducted by the endocrinologists and objective assessments could be due to the model being insensitive to other clinical factors adding to the complexity of the management of the disease and care of the patient with T2D. The model could be more sensitive if relevant co-morbidities were incorporated into the model, as it was allegedly the most frequent reason for the endocrinologists to allocate patients to a higher level compared to the objective assessment in the present study. Information on major cardiovascular events (MACE), symptomatic cardiovascular disease and heart failure was integrated in our model, but other clinical relevant co-morbidities could be relevant as well. One study integrated co-morbidities as a separate parameter in the model [17] and defined it as severe co-morbidity requiring another disease-specific management than the endocrinologist [17]. This definition is very broad as it will include co-morbidities not being relevant to T2D, and thereby allocate patients to higher risk levels. If co-morbidities are going to be incorporated in the model, it must be defined in a clear and precise way.

Blood pressure was one of the most frequent reasons for the objective assessment being higher than the endocrinologists, as endocrinologists tended to underestimate the value of the blood pressure in the categorisation of stratification level; therefore the thresholds for blood pressure and the circumstances under which it was measured should be discussed. Blood pressure measured at the doctor’s office, even after 5–10 min of rest, may still be higher than the ‘real’ value, for instance due to white coat hypertension. A recent review found that monitoring 24-h ambulatory blood pressures was a better predictor of cardiovascular events than office blood pressure levels. In terms of blood pressure control in patients with T2D, the authors recommended a more frequent usage of 24-h ambulatory blood pressure monitoring [32]. Furthermore, the Danish risk stratification model can be discussed as the defined thresholds did not take individual treatment goals into account. For instance, the glycaemic target is often more stringent for patients with T2D only treated with metformin and lifestyle changes and less stringent for patients with more advanced micro or macrovascular diseases or extensive co-morbidities [20]. This indicates that the model can be used as guidance in the risk stratification performance, but the clinical assessment performed by the endocrinologists will be conclusive for the actual risk level allocation of the individual patient.

Even though the present study found discrepancies in one third of the assessments, a modified version of the model might be useful in identifying patients at risk. The substantial number of disagreements might be reduced if an electronic decision support system calculating an estimated stratification level was available during the consultation for the endocrinologist. Electronic support systems for generating risk stratification levels have been developed and tested in ambulatory diabetes care [30, 33] as well as in primary care clinics [34–36]. The Joined Asian Decision Evaluation (JADE) program was validated by following a cohort, and after a median follow-up period of 5.5 years it was shown that higher risk levels were associated with increased risk of clinical endpoints such as CVD, end-stage renal disease and death [30]. In addition to risk stratification, the JADE program produces a care protocol with predefined management plans and schedules for follow-up to support decision making and promote the interaction between the patient and the healthcare professional [30, 33]. The program tested in primary care clinics in USA is extended with suggestions for regulation of treatment [34]. While the Asian model [30, 33] incorporated multiple parameters for generating a risk level for the patient, the American model [34] risk stratified for each parameter. The latter is similar to the diabetes-specific database already used in our clinic

Future research should investigate whether or not a risk stratification model is a relevant and useful tool in the organisation of the growing number of patients with T2D. The model used in the present study recommends that patients should be assigned to a speciality level in accordance with increasing severity and complexity of the disease, and personal abilities [16, 37]. The majority of patients in our study seemed to be at medium or high risk levels where emerging complications could progress if proper and medium-level specialty care is not given [9, 11, 18, 19]. It seems to be the case that some of these patients are currently controlled in primary care and some are controlled at a specialised level [13]. Therefore, it would be relevant to investigate if this group of patients, at the medium-risk level, would benefit from a shared care follow-up, where the general practitioners have the responsibility for the diabetes care and treatment and the regular visits at the general practitioners office are combined with an annual extensive check-up in a specialised diabetes outpatient clinic. Such an intervention could offer highly specialised diabetes care and at the same time diluting the care burden between general practitioners and hospital-based outpatient clinics.

To our knowledge, this is the first study investigating the degree of agreement between clinical and objective assessments by the use of a risk stratification model for patients with T2D. Furthermore, the present study included a large group of patients, who were unselected and included consecutively. The objective assessments of risk stratification levels were conducted according to a well-defined model [16].

The present study had some limitations. Initially we included all patients with T2D followed in the outpatient clinic. However, some patients, especially those in the long-term follow-up, did not have an annual extensive check-up performed and had to be excluded from the study since risk stratification was meant to be performed at this visit according to our clinic visit schedule. This is a bias in our study, as this group may represent a certain type of patients; e.g. patients with irregular visiting patterns at the clinic as well as instability regarding self-care. Reasons for the mismatches in risk stratification assessments could also be that not all data were available for the endocrinologist at the time of visit, for instance test results from urine and blood samples taken the same morning as the visit. However, this is considered not to be influencing the overall results of the study, as it rarely occurs. Another limitation is that this is a single center study. Including patients from multiple diabetes outpatient clinics could enhance the external validity of the results.

## Conclusions

In conclusion, the endocrinologists were generally compliant in performing the risk stratification, and the population allocated for long-term specialised follow-up was indeed patients with medium or high risk and complexity in accordance with guidelines Our data suggest an opportunity for decision support to improve adherence to the Danish risk stratification guidelines. It might offer guidance for the organisation of future diabetes care in general practice, specialised diabetes outpatient clinics and improved cooperation between these caregivers.

## Abbreviations

BMI: body mass index
CVD: cardiovascular disease
DBP: diastolic blood pressure
HbA_1c_: haemoglobin A_1c_
HDL: high density lipoprotein
LDL: low density lipoprotein
MACE: major cardiovascular event
NYHA: the New York Heart Association functional classification in patients with heart disease.
SBP: systolic blood pressure
T2D: type 2 diabetes
TC: total cholesterol
TG: triglycerides
